# Stenotrophomonas maltophilia Endocarditis of an Implantable Cardioverter Defibrillator Lead

**DOI:** 10.7759/cureus.4165

**Published:** 2019-03-01

**Authors:** Mohan Satish, Muhammad Adil Mumtaz, Marvin J Bittner, Carrie Valenta

**Affiliations:** 1 Internal Medicine, Creighton University Medical Center, Omaha, USA; 2 Hospital Medicine, Creighton University Medical Center, Omaha, USA

**Keywords:** s. maltophilia, infective endocarditis, nosocomial

## Abstract

*Stenotrophomonas maltophilia* (*S. maltophilia*) is a nosocomial pathogen and a rare cause of infective endocarditis (IE). Given the intrinsic resistance to many classes of antibiotics, IE due to *S. maltophilia* carries significant morbidity and mortality among the cases described. Prompt identification of risk factors, particularly the use of medical devices, is necessary for the timely identification of this organism and prompt medical management. We report a case of an implantable cardioverter defibrillator (ICD) lead associated IE due to *S. maltophilia* and discuss the diagnosis, treatment and outcomes in relation to existing evidence.

## Introduction

*Stenotrophomonas maltophilia* (*S. maltophilia*) is an aerobic, Gram negative bacillus and opportunistic pathogen. *S. maltophilia* has been mainly associated with nosocomial infections from contaminated medical equipment (e.g., central venous or urinary catheters, mechanical ventilators) or recent surgery [1-3]. Its growing emergence within the immunocompromised population has been of concern given that it is resistant to several classes of antibiotics [4-6]. While typically* S. maltophilia* has been associated with bacteremia and pneumonia, infective endocarditis (IE) is quite rare and associated with significant morbidity and mortality among the 45 reported cases known to date worldwide [4,7-9]. Specifically, implantable cardioverter defibrillator (ICD) or pacemaker-IE due to *S. maltophilia* has only been reported in one of these cases [10]. Here, we describe a case of ICD lead-IE due to *S. maltophilia* in the setting of a patient with end stage renal disease (ESRD) with need for a permanent dialysis catheter.

## Case presentation

A 53-year-old African-American woman with ESRD was transferred from dialysis clinic to the emergency room (ER) for evaluation of non-radiating and dull epigastric pain for two weeks associated with fever and chills during hemodialysis (HD). Three months ago, she was hospitalized and treated for *Streptococcus pneumoniae *and *Enterobacter cloacae* bacteremia. A year ago she was treated for *S. maltophilia *bacteremia secondary to an infected dialysis catheter. Past medical history was also significant for hypertension, atherosclerotic vascular disease pending elective coronary artery bypass graft (CABG) surgery, and sudden cardiac arrest followed by ICD placement. With the current presentation, both blood and catheter cultures obtained at the dialysis clinic were positive for *S. maltophilia*, prompting her subsequent arrival to the emergency room. She presented with a continuation of fever and chills, as well as tachycardia and episodic hypotension. She was noted to have mild epigastric tenderness. There was no surrounding erythema, discharge, or tenderness noted around the tunneled dialysis catheter on the right anterior chest. Initial workup showed elevated troponin and procalcitonin. Chest X-ray findings were suggestive for left lower lobe pneumonia. Within the ER, the patient's hypotension resolved with fluid resuscitation and intravenous levofloxacin therapy was started with blood cultures drawn.

Cardiology was consulted for persistent elevation of troponins and it was presumed secondary to impaired clearance in ESRD. Transthoracic echocardiography (TTE) was done to evaluate for endocarditis given the presentation of bacteremia and fevers. TTE revealed artifact noted on an abandoned ICD lead in the right heart concerning for possible vegetation. Repeat blood cultures were positive for *S. maltophilia* and the patient was continued on levofloxacin. Infectious Diseases was consulted and as per their recommendation the infected tunneled dialysis catheter was removed on the 2nd day of the hospitalization (DOH). The patient continued to be febrile despite levofloxacin therapy and a transesophageal echocardiogram (TEE) was performed to look for a cardiac source. Subsequent blood cultures were negative at this time but the patient continued to be symptomatic. The TEE was done on the 4th DOH and showed a 1 x 0.5 cm echodensity attached to an abandoned right ventricular (RV) ICD lead in the superior vena cava (SVC) as it entered into the right atrium (RA). The echodensity was concerning for a vegetation due to IE. ID consultation recommended removal of the abandoned lead with culture of the probable vegetation that may have served as a nidus for recurrent bacteremia. Levofloxacin therapy was continued and a new dialysis catheter was placed on the 5th DOH with HD restarted. At this time, the patient became afebrile and reported symptomatic improvement. Despite a strong suspicion for IE by Duke criteria, fluorodeoxyglucose positron emission tomography (FDG PET) on the 6th DOH was non-diagnostic for differentiating infective etiology from thrombotic. Cardiothoracic surgery was consulted for removal of the abandoned lead for culture and planned to do so in coordination with her pending elective CABG procedure. In the interim, repeat blood cultures remained negative on the 7th DOH and the patient was stable and discharged on the 12th DOH with instructions to transition to oral levofloxacin until her CABG procedure, scheduled 18 days from discharge.

Unfortunately, the patient expired due to complications from cardiac arrest secondary to severe hypokalemia in the postoperative period after removal of the infected ICD lead and successful CABG. No vegetation could be appreciated on gross inspection of the removed ICD-lead and subsequent culture was negative for any growth, indicating a resolution of the IE over the one-month course of levofloxacin treatment since presentation.

## Discussion

To our knowledge this is the second report of pacemaker- or ICD-lead IE due to *S. maltophilia*, and 46th case of IE due to *S. maltophilia *of any cause. By Duke criteria, the presence of two separate blood cultures for *S. maltophilia* (major criteria), and the patient’s febrile presentation (minor criteria) alone made IE probable in this case. Despite a lack of typical physical exam findings, subsequent TEE evidence of a possible vegetation (major criteria) in the setting of recurrent bacteremia due to *S. maltophilia* in this patient strongly suggested IE. Furthermore, the patient experienced a rapid resolution of her fever on the 2nd DOH upon initiation of levofloxacin treatment with subsequent blood cultures being negative. As she was continued on levofloxacin for nearly a month, the clearance of the observed vegetation on the removed ICD-lead in addition to negative lead cultures suggested an excellent response to ongoing treatment.

**Figure 1:**
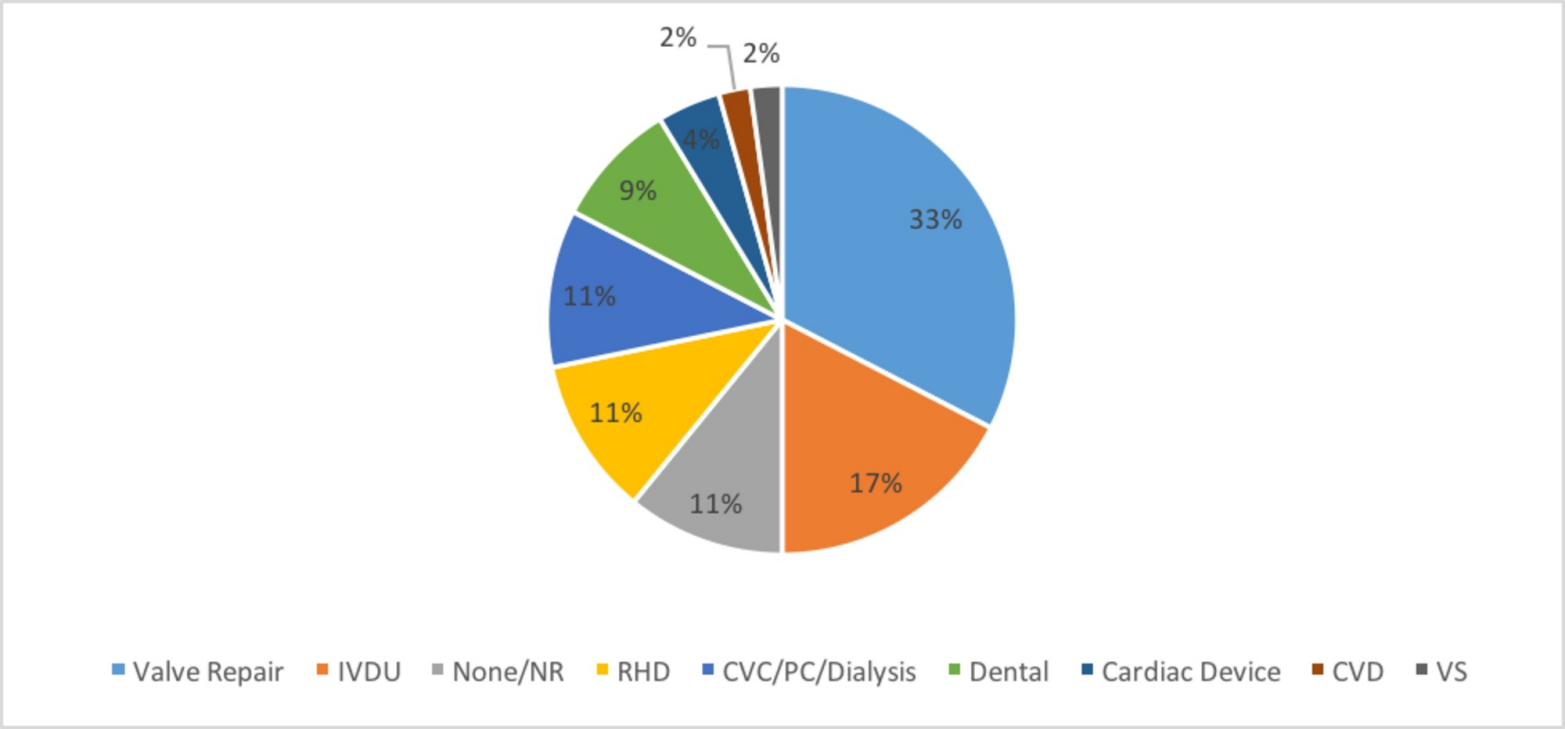
Risk Profile in Infective Endocarditis due to Stenotrophomonas maltophilia. Risk factors noted in cases of infective endocarditis due to *Stenotrophomonas maltophilia* (n = 46 cases) by percent weight. Adapted and updated from Subhani et al. [7]. Valve Repair - Cardiac valve repair; RHD - Rheumatic heart disease; Cardiac Device - Pacemaker/ICD; IVDU - Intravenous drug use; CVC/PC/dialysis - Central venous catheter/peripheral catheter/dialysis; CVD - Collagen vascular disease; Dental - Dental treatment; VS - Ventriculoatrial shunt; None/NR - No risk/not reported.

Despite the successful resolution of IE noted in this case, IE due to *S. maltophilia* is associated with a high mortality rate (33%) in the previous cases reported [7]. Irrespective of complications that contributed to mortality, morbidity in general was higher in these cases with myocardial abscess, cerebral infarction, and congestive heart failure noted among 70 to 80% of these causes [7]. Early identification in this case may explain the lack of typical physical examination findings (e.g., Janeway lesions, Osler nodes, etc.) and preventing the progression to complications. Nevertheless, the presence of IE related to an abandoned lead from the patient’s old ICD is a unique concern among the noted risk factors (Figure 1). In general, pacemaker- or ICD-lead related IE is a rare phenomenon and related to predisposing factors such as intravenous catheters, age, and diabetes mellitus [11-12]. In the case presented here, the patient’s need for a permanent tunneled dialysis catheter provided an infectious nidus with recurrent bacteremia and the hematogenous spread of *S. maltophilia *to the abandoned ICD-lead. In the previous case of pacemaker-related IE due to *S. maltophilia*, the infection was initially recalcitrant to antibiotic therapy requiring removal of the entire pacemaker system before a successful antibiotic regimen. Notably, the patient was 19 years older (72-year-old) and had a history of intermittent antibiotic therapy for chronic middle and external otitis [10]. Furthermore, it has been shown that infection of an abandoned cardiac device lead requires aggressive management and is associated with an increased need for laser extraction compared to cardiac device infections without an abandoned lead [13]. Although complicating management, cardiac device lead extraction itself is associated with complication rates close to 3% and a mortality rate of 34% among these complications [14]. Therefore, the vulnerability to an infection with a pathogen like *S. maltophilia *must be assessed in committing to such implantable cardiac devices, where antibiotic therapy may be the only option. For example, recent evidence has implicated autoimmune disease as a predisposing factor for IE due to *S. maltophilia* [15].

Previously, the rapid response to antimicrobial treatment of* S. maltophilia *caused IE has been noted with fluoroquinolone monotherapy. Fluoroquinolone monotherapy has been found to have comparable efficacy to trimethoprim-sulfamethoxazole (TMP-SMX) but is associated with rapid development of resistance upon use [16-17]. Fluoroquinolones are known to have bactericidal activity against *S. maltophilia* compared to the bacteriostatic activity of TMP-SMX [18-19]. Although TMP-SMX is considered the first-line choice for *S. maltophilia* infections, its use was contraindicated due to this patient’s ESRD. Her repeat response to levofloxacin was a very fortunate outcome with a recurrent monomicrobial *S. maltophilia* infection. Given that antimicrobial treatment directed against *S. maltophilia* has been a challenge due to intrinsic resistance to several classes of antibiotics, evidence of repeat resolution with levofloxacin monotherapy is rare. Given the common occurrence of nosocomial infections with *S. maltophilia* on medical devices, it is necessary to note that the prompt removal of infected foreign material is also critical if feasible. Nevertheless, the early identification of this infection with this pathogen and evaluation of its antibiotic susceptibility was most likely responsible for the success in this case, especially considering the patient’s risk profile.

## Conclusions

With the growing emergence of nosocomial infections due to *S. maltophilia* due to intrinsic antibiotic resistance, IE is a rare but serious concern fraught with significant morbidity and mortality. The case reported here is among a limited set of cases illustrating the timely identification and medical management of IE caused by *S. maltophilia*.
